# Retina-attached slice recording reveals light-triggered tonic GABA signaling in suprachiasmatic nucleus

**DOI:** 10.1186/s13041-021-00881-9

**Published:** 2021-11-27

**Authors:** Jea Kwon, Minwoo Wendy Jang, C. Justin Lee

**Affiliations:** 1 KU-KIST Graduate School of Converging Science and Technology, Korea University, 145 Anam-ro, Seongbuk-gu, Seoul, 02841 Republic of Korea; 2 Center for Cognition and Sociality, Institute for Basic Science (IBS), 55 Expo-ro, Yusung-gu, 34126 Daejeon, Republic of Korea

**Keywords:** Whole-cell patch, Spatiotemporal analysis, Retina-attached SCN, Light triggered tonic GABA

## Abstract

Light is a powerful external cue modulating the biological rhythm of internal clock neurons in the suprachiasmatic nucleus (SCN). GABA signaling in SCN is critically involved in this process. Both phasic and tonic modes of GABA signaling exist in SCN. Of the two modes, the tonic mode of GABA signaling has been implicated in light-mediated synchrony of SCN neurons. However, modulatory effects of external light on tonic GABA signalling are yet to be explored. Here, we systematically characterized electrophysiological properties of the clock neurons and determined the spatio-temporal profiles of tonic GABA current. Based on the whole-cell patch-clamp recordings from 76 SCN neurons, the cells with large tonic GABA current (>15 pA) were more frequently found in dorsal SCN. Moreover, tonic GABA current in SCN was highly correlated with the frequency of spontaneous inhibitory postsynaptic current (sIPSC), raising a possibility that tonic GABA current is due to spill-over from synaptic release. Interestingly, tonic GABA current was inversely correlated with slice-to-patch time interval, suggesting a critical role of retinal light exposure in intact brain for an induction of tonic GABA current in SCN. To test this possibility, we obtained meticulously prepared retina-attached SCN slices and successfully recorded tonic and phasic GABA signaling in SCN neurons. For the first time, we observed an early-onset, long-lasting tonic GABA current, followed by a slow-onset, short-lasting increase in the phasic GABA frequency, upon direct light-illumination of the attached retina. This result provides the first evidence that external light cue can directly trigger both tonic and phasic GABA signaling in SCN cell. In conclusion, we propose tonic GABA as the key mediator of external light in SCN.

**Supplementary Information:**

The online version contains supplementary material available at 10.1186/s13041-021-00881-9.

## Introduction

Light is a strong external cue which can powerfully modulate circadian rhythm in mammalian animals. Light input received from the retina is transmitted as electrical signals to SCN through the retinohypothalamic tract (RHT) [1]. Neurotransmitters released from optic nerve, such as glutamate, are thought to synchronize SCN neurons and mediate light-induced changes [2]. Among various molecular substrates, GABA has been proposed as one of the major downstream target for light-mediated circadian rhythm changes [3–6].

Despite the general consensus on the importance of GABA in SCN, the role of GABA has been controversial over the question of whether it synchronizes or desynchronizes SCN neurons [6]. This discrepancy is the result of a variety of confounding factors including different activity pattern across the circadian period, heterogeneity of cell types across the regions, different modes (phasic and tonic) of GABAergic actions, switching of inhibitory to excitatory action of GABA and so on [6]. Two previous back-to-back publications have addressed this complex nature of GABA in SCN by both mathematical modeling and experimental observations [5, 7]. Myung et al. have shown that a different length of daily light-exposure can significantly affect the coupling of dorsal and vental SCN neurons via GABA signaling [5]. Furthermore, simulation results emphasized that tonic but not phasic GABA is critical for synchrony between SCN neurons [7]. Their results raise the possibility that the light-mediated dynamics of GABA signaling is divergent across the time and region. However, empirical evidence has been lacking.

Experimentally, whole-cell slice recording is regarded as a gold standard for investigation on phasic and tonic GABA signaling, because these events occur at sub-second level [8]. Although the role of tonic GABA has been widely characterized in various brain regions and cell types [9–14], electrophysiological profiling of tonic GABA in SCN is still insufficient. Recently, Moldava et al. reported the existence of diurnal rhythms in tonic GABA, mediated by spill-over from presynaptic acitvities in SCN [15]. However, whether external-light cue is involved in tonic GABA signaling remains elusive.

In this study, we focused on systematically characterizing tonic GABA signaling in SCN, while considering not only temporal but also spatial information. In addition, we sought for experimental evidence that GABA signaling can be triggered upon direct light-illumination, by performing retina-attached SCN slice recordings.

## Materials and methods

### Animals

BALB/c male mice of postnatal day from 50 to 96 were used for ex-vivo slice patch clamping experiments. For training of retina-attached slice preparation, we used 8 weeks old C57BL/6J mice. all animals were housed in 12:12 light-dark cycle.

### Recording conditions and solutions for electrophysiology

We took advantage of N-Methyl-D-glucamine (NMDG) protective recovery method for high quality of slice preparation [16]. For protective cutting solution, we used NMDG solution (93 mM NMDG, 93 mM HCl, 2.5 mM KCl, 1.2 mM NaH$_2$PO$_4$, 30 mM NaHCO$_3$, 20 mM HEPES, 25 mM Glucose, 5 mM sodium ascorbate, 2 mM Thiourea, 3 mM sodium pyruvate, 10 mM MgCl$_2$, 0.5 mM Cacl$_2$, 12 mM N-Acetyl Cystein, and pH-adjusted to 7.4). For recording solution, we used aCSF solution (130 mM NaCl, 3.5 mM KCl, 24 mM NaHCO$_3$, 1.25 mM NaH$_2$PO$_4$, 1.5 mM CaCl$_2$, 1.5 mM MgCl$_2$, 10 mM d-(+)- glucose, and pH-adjusted to 7.4). All external solutions were saturated with 95% O$_2$ and 5% CO$_2$ more than 30 min. For spatiotemporal profiling of GABA signaling, we used CsCl-based internal solution (135 mM CsCl, 4 mM NaCl, 0.5 mM CaCl$_2$, 10 mM HEPES, 5 mM EGTA, 2 mM Mg-ATP, 0.5 mM Na$_2$-GTP, 10 mM QX-314, and pH-adjusted to 7.2 with CsOH). We performed voltage clamp recording to measure the chloride currents (held at −60 mV). To exclude the excitatory signals, the baseline current was stabilized with D-AP5 (50 $\mu$M) and CNQX (20 $\mu$M). For retina-attached SCN slice patch, we used CsMeSO$_4$-based internal solution (120 mM CsMeSO$_4$, 5 mM NaCl, 4 mM CsCl, 10 mM HEPES, 2 mM EGTA, 4 mM Mg-ATP, 0.3 mM Na-GTP, and 7 mM Tris$_2$-phosphocreatine and were adjusted to pH 7.3 with CsOH, see [17]). We recorded inhibitory post-synaptic current by clamping the voltage at 0 mV, in Fig. 2, to selectively obtain GABA current without using pharmacological blockers of glutamate receptors to avoid blockade of RHT released glutamat signals.

### General slice preparation procedure

Before starting the surgical procedure, 150 ml of NMDG-based protective cutting solution was ice-chilled (0–2$^\circ \hbox {C}$) for both transcardial perfusion and brain slicing. Also, 150 ml of NMDG-based protective cutting solution was heated (28–32$^\circ \hbox {C}$) in chamber for initial recovery. Recording aCSF solution was put in room temperature for additional recovery. After preparation of all solutions and apparatus, mice were deeply anesthetized with 2% avertin (20 mg g$^{- 1}$, intraperitoneally) and transcardial perfusion was done manually with 20 ml NMDG-based external solution. After decapitation, the brain was quickly excised from the skull and submerged in ice-cold NMDG recovery solution (for retina-attached brain extraction, see retina-attached SCN slice preparation section). When mice were decapitated during the dark period, the procedures were conducted in a dark room under dim light illumination. The hemisected brain was glued onto the stage of a vibrating microtome (PRO7N; DSK) and 250 $\mu$m-thick slices were cut and transferred to initial recovery solution (10–12 min, 28–32$^\circ \hbox {C}$). After the initial recovery period, slices were transferred to normal aCSF solution for additional recovery (>20 min, room temperature). Note that we obtained one or two slices from one animal, and multiple anesthetized mice (up to 5 of littermates) were sliced at a time.

### Retina-attached SCN slice preparation

**Fig. 1:**
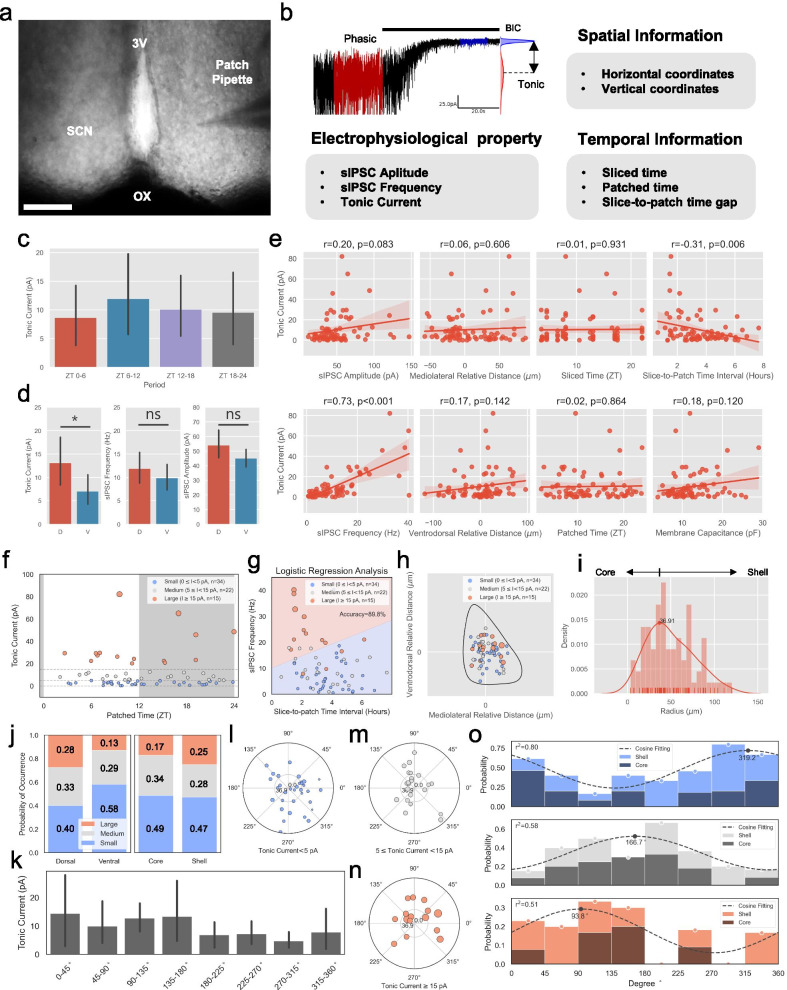
Spatio-temporal profiling of tonic GABA in SCN. **a** Differential interference contrast (DIC) image of freshly isolated mouse brain with glass pipette whole-cell patch clamped to a neuron. 3V; third ventricle, SCN; Suprachiasmatic nucleus, OX; Optic chiasm. Scale bar: 100 $\mu$m **b** Representative trace of tonic GABA current recording in a SCN neuron and list of information we gathered **c** Summary bar graphs of tonic current analyzed by period categories. Bar graphs are shown as mean and error of 0.95 confidence intervals. **d** Summary bar graphs of dorsal and ventral tonic current (left), sIPSC Frequency (middle), sIPSC Amplitude (right). Bar graphs are shown as mean and error of 0.95 confidence intervals. **e** Pearson’s correlation analysis of tonic current with sIPSC amplitude, sIPSC frequency, mediolateral relative distance, ventrodorsal relative distance, sliced time, patched time, slice-to-patch time interval and membrane capacitance. **f** Tonic current scatter plot on patched time grouped by tonic current size: “Small” (blue), “Medium” (gray), “Large” (red). Dashed lines indicate decision boundaries for three groups. Dark shaded area, ZT 12–24. **g** Logistic regression analysis with “Large” and “Small” group with sIPSC frqeuncy and slice-to-patch time interval. A line divided by blue and red areas represent decision boundary with accuracy of 89.8%. **h** Tonic current scatter plot on 2D virtual SCN map, colors and size indicates group and size of tonic current respectively. **i** Histogram and gaussian kernel density estimation(KDE) on radius axis from SCN center. SCN center is calculated by 2d centroid of virtual SCN area. Core and shell was divided at the peak of KDE. **j** Probability of occurrence of each group in dorsal or ventral (left) and core or shell (right). **k** Summary bar graph of topologically divided groups of tonic current. **l, m** Scatter polar charts of tonic current groups. 0$^\circ$, 90$^\circ$, 180$^\circ$ and 270$^\circ$ respectively indicates lateral, dorsal, medial and ventral directions. Group “Small” ($0\le {I}<5$ pA, (l) n = 34); Group “Medium” ($5\le {I}<15$ pA, n = 22) (m), “Large” ($I>15$ pA, n = 15) (**n**) **o**, Probability of occurrence and cosine fitting in 8 different radial divisions: Group “Small”, regression coefficient r$^2$=0.80, phase shift $\theta$ = 319.2$^\circ$ (top); Group “Medium”, regression coefficient r$^2$ = 0.58, phase shift $\theta$=166.7$^\circ$ (middle); Group “Large”, regression coefficient r$^2$ = 0.51, phase shift $\theta$ = 93.8$^\circ$ (bottom).

This procedure required repeated trials and errors during training to obtain desirable quality of slice. We optimized some of the steps of original retina-attached slice method to improve slice quality [17]. Most important difference from the original protocol was to use ice-chilled NMDG protective method throughout the slicing since it slowed down the metabolism via immediate cooling of brain through transcardial perfusion and reduced excitotoxicity by low Na$^{+}$, low Ca$^{2+}$/high Mg$^{2+}$. As trained experimenter required about 40 minutes of surgical operation time, this process significantly helped improving slice quality. Therefore, we followed NMDG protective procedure up to decapitation (see general slice preparation procedure). After decapitation, we performed retina-attached brain extraction procedure (Fig. 1a): (1) Remove all scalps and lower jaw was removed by making a horizontal cut with bone cutting scissors; (2) Cut the occipital bone; (3) Cut both of the zygomatic bones; (4) Remove the skull cap by cutting along the base of the calvarium bilaterally; (5) Cut the nasal and frontal bones with razor blade vertically; (6) Remove the remaining surrounding bones nearby the plate bone; (7) Cut the plate bone to expose the space between brain and optic nerve fixed on the remaining bone; (8) Using microscissors, carefully cut away all remaining bones and connective tissue to isolate the brain and eyes as a unit in ice-cold NMDG solution; (9) To expose retina, get rid of sclera, choroids, and pigment epithelium by fine-forceps and microscissors. (for details on retina exposure, see [18]). Next, the hemisected brain is glued onto the stage of a vibrating microtome. While slicing, cautiously position the retina attached optic nerves with blunt forceps to prevent cutting from blades. Slice near the SCN with thickness of 600 $\mu$m, which is connected with optic nerves. Put isolated retina-attached SCN slice to recovery NMDG solution (28–32$^\circ \hbox {C}$) for 10–12 min. Next, transfer slice to normal aCSF solution chamber for more than 20 min. Both recovery chamber and normal aCSF chamber are maintained under darkness.

### Light-illumination on retina-attached SCN

Our experimental design was to mimic photo stimulation protocol used in phase-delay experiment in animal circadian rhythm research. When slice is positioned at recording chamber of the patch rig, retina should be positioned away from recording site to minimize the light exposure during patch. Retina should face directly to the light source. As dark adaptation has been pointed out to be critical for light-induced responses in retina-attached SCN [17], slice was remained for 30 min of dark adaptation after forming a configuration of whole cell. After dark adaptation, we started recording and waited for 10 min of stable baseline. For photo stimulation, we applied ambient light (300–400 lux, measured by luminometer) on retina for duration of 15 min. Recovery period was followed by more than 10 min. Whole cell was maintained for about 70 min including dark adaptation and total recording duration was about 40 min.

### Hardware and software

The slice chamber was mounted on the stage of an upright microscope and viewed with a 60X water immersion objective (numerical aperture = 0.90) with infrared differential interference contrast optics. Cellular morphology was visualized by a complementary metal oxide semiconductor camera and the Imaging Workbench software (INDEC BioSystems, ver. 9.0.4.0.). Electrical signals were digitized and sampled at 10 ms intervals with Digidata 1550 data acquisition system and the Multiclamp 700B Amplifier (Molecular Devices) using the pClamp10.2 software. Data were filtered at 2kHz. For ambient light stimulation(300–400 lux measured by portable luminometer) we used Leica CLS 150 X Microscope Cold Light Source. 3D mouse skull images were obtained by resources from http://digimorph.org/specimens/Mus_musculus/.

### Data analysis

We logged all necessary information for spatiotemporal analysis (see raw data [see Additional file 2]). We used python and related libraries for acquired data analysis. Some off-line statistical analysis was carried out using Clampfit version 10.4.1.10 and GraphPad Prism version 7.02 software. The frequency and amplitude of phasic GABA currents before bicuculline administration was detected and measured by Mini Analysis (Synaptosoft, ver. 6.0.7.). For tonic GABA analysis, we obtained mode values from fitted histograms of 1 min length traces before and after bicuculline administration. When comparing between two samples, significance of data was assessed by Mann-Whitney test because samples did not pass normality test. When comparing more than 2 samples, significance of data was assessed by one way ANOVA with Kruskal-Wallis test with Dunn’s post-hoc test because the data did not pass normality test. Significance levels were given as: N.S. $p > 0.05$, *$p < 0.05$, **$p < 0.01$, ***$p < 0.001$ and #$p < 0.0001$.

In categorical analysis, we used linear discriminant analysis (LDA) which addresses continuous independent variables of slice-to-patch time interval and sIPSC freqeucny and a categorical dependent variables of “Small” and “Large” tonic current groups.

1 $$\begin{aligned} \text {Accuracy} = \frac{\text {Number of correct predictions}}{\text {Total number of predictions}} \end{aligned}$$

In radial distribution analysis, we measured probability of occurrences in each radial divisions with “Small”, “Medium” and “Large” categorical groups. Next, cosine fitting was performed to detect maximal angle. Given that *x* is bin center of radial division, $\hat{y}$ is predicted probability of occurrence, we fitted below equation.

2 $$\begin{aligned} \hat{y} = A\cos {(x-\theta )}+b \end{aligned}$$

Three parameters, *A* is (Amplitude), *b* is (bias) and $\theta$ is (angle), were fitted with least square objective function shown below.

3 $$\begin{aligned} \text {min}\sum _{n=1}^{n}{(\hat{y_i}-y_i)^2} \end{aligned}$$

### Assignment of horizontal and vertical coordinates

To assign relative coordinate of patched cells from center of SCN, we took low resolution DIC images after recording and manually drew the boundaries of SCN. Then we compared with reference SCN boundaries from Allen brain atlas map [19]. For SCN reference map, we calculated centroid of given 2D area, and used as a center of relative coordinates when we assign relative coordinates (Additional file 1: Fig. S1).

## Results

### Spatio-temporal profiling of tonic GABA in SCN

The previous computational modeling predicted that the heterogeneity of spatial and temporal diversity in SCN might have differential effect on GABA signaling [7]. Furthermore, spill-overs from the high frequency phasic inputs are thought to be a determinant for extracellular GABA level in SCN [6, 15]. To investigate the factors that regulate the extracellular GABA level in SCN, we performed a whole-cell patch clamp recording in 76 cells (from conventional slice preparation method) and carefully structured spatio-temporal profiles. For each cell, we systematically measured tonic current, sIPSC amplitude, sIPSC frequency, sliced time (the time of decapitation), patched time, slice-to-patch time interval (the time interval between sliced time and patched time), coordinates of ventrodorsal and mediolateral axis (Fig. 1a, b, for raw data [see Additional file 2]). With this dataset, we conducted a basic categorical analysis using a broadly used criteria [20–24]. We divided 76 cells into 4 groups based on zeitgeber time (ZT) of sliced time: ZT 0–6, ZT 6–12, ZT 12–18, ZT 18–24. Our result did not show any statistical difference among temporal groups (ZT 0–6, 8.65 ± 2.67 pA; ZT 6–12, 11.94 ± 3.81 pA; ZT 12–18, 10.06 ± 2.75 pA; ZT 18–24, 9.52 ± 3.63 pA; mean ± s.e.m) (Fig. 1c). For regional analysis, we divided SCN into dorsal and ventral regions. We found significantly larger tonic current in dorsal neurons compared to ventral neurons (p = 0.041, Dorsal vs Ventral; Dorsal, 13.09 ± 2.75; Ventral, 7.00 ± 1.66; mean ± s.e.m), but not in sIPSC amplitude (p = 0.606, Dorsal vs Ventral; Dorsal, 13.09 ± 2.75; Ventral, 7.00 ± 1.66; mean ± s.e.m) and frequency (p = 0.462, Dorsal vs Ventral; Dorsal, 13.09 ± 2.75; Ventral, 7.00 ± 1.66; mean ± s.e.m) (Fig. 1d). To explore the relationship between tonic current and other features, we conducted a Pearson’s correlation analysis on various factors with tonic GABA current to extract meaningful data (Fig. 1e). In terms of relationship between tonic and phasic GABA, we found a positive correlation on tonic current with sIPSC frequency and amplitude (Pearson’s Coeff. of sIPSC Frequency, r = 0.73, p<0.001, n = 76; sIPSC Amplitude, r = 0.20, p = 0.083, n = 76). High correlation between tonic GABA current and sIPSC frequency was consistent with the previous report [15]. For spatial analysis, ventrodorsal axis showed higher positive correlation compared to mediolateral axis with tonic current (Pearson’s Coeff. of Mediolateral Relative Distance, r = 0.06, p = 0.606, n = 76; Ventromedial Relative Distance, r = 0.17, p = 0.142, n = 76). Although temporal analysis on sliced time or patched time did not show any correlation (Pearson’s Coeff. of Slice Time, r = 0.01, p = 0.932, n = 76; Patched Time, r = 0.02, p = 0.864, n = 76), interestingly, slice-to-patch time interval, showed relatively high negative correlation (Pearson’s Coeff. of Slice-to-patch time interval, r = − 0.31, p = 0.006, n = 76). To investigate whether this gradual decrease in extracellular GABA level along with the elapsed time after slicing is caused by deterioration of metabolic health of brain slices, we analyzed cell input and series resistances of our recording condition. However, our recording condition did not vary considerably with slice-to-patch time (Pearson’s correlation coefficient of slice-to-patch time interval with series resistance, r = 0.135, p = 0.245, n = 76; slice-to-patch time interval with input resistance r = 0.080, p = 0.492, n = 76; series resistance with tonic current, r = − 0.102, p = 0.381, n = 76; input resistance with tonic current, r = − 0.055, p = 0.637 , n = 76) (Additional file 1: Fig. S2a–d), raising another possibility that retinal light exposure in intact SCN is critical for generation of tonic GABA current in SCN.

To investigate spatial pattern of tonic current thoroughly, we arbitrarily established a criteria for categorizing the cells based on the level of tonic GABA currents: “Small” ($0\le {I}<5$ pA, n = 34), “Medium” ($5\le {I}<15$ pA, n = 22), “Large” ($I>15$ pA, n = 15) (Fig. 1f). With these criteria, we conducted basic property analysis and revealed that “Large” group significantly differs in sIPSC frequecny, sIPSC amplitude and slice-to-patch time interval compared to “Small” group. (Additional file 1: Fig. S3f). Moreover, As correlation analysis revealed sIPSC freqeuncy and slice-to-patch time interval as meaningful features, we conducted logistic regression classification analysis with “Large” and “Small” groups to examine whether those features can manifest the level of tonic GABA current. The result showed the classification accuracy of $89.8\%$ indicating that these two factors powerfully manifest the tonic GABA currents (Fig. 1g). Next, we visualized these groups on the virtual SCN map with exact mediolateral and ventrodorsal coordinates. Our results clearly show a clam shell-like pattern of amplitude distribution of tonic GABA currents (Fig. 1h). To quantitatively analyze spatial pattern of tonic GABA in SCN, we first divided core and shell population based on Gaussian kernel density estimation on radius axis (Core, $r\le 36.91$; Shell, $r>36.91$) (Fig. 1i). Based on this criteria we found that “Large” tonic current groups are frequently found in dorsal, shell region (Dorsal Large, p = 0.28; Ventral Large, p = 0.13; Core Large, p = 0.17, Shell Large, p = 0.25) (Fig. 1j). Next, we introduced topological analysis approach [25], and found that tonic current exhibit a moderate trend of larger current size in a range of 0$^\circ$–180$^\circ$ (Fig. 1k). By exploring group-wise radial distribution, we discovered that “Small” populations are mostly located at ventro-lateral region ($\theta$=319.2$^\circ$) whereas “Large” populations reside in dorsal region ($\theta$=93.8$^\circ$) (Fig. 1l–o). Together, our electrophysiologcal characterization revealed that dorsal-shell neurons with high sIPSC frequency and short slice-to-patch time interval are likely to have high tonic current.

### Light triggered sustained tonic GABA signaling in SCN

**Fig. 2:**
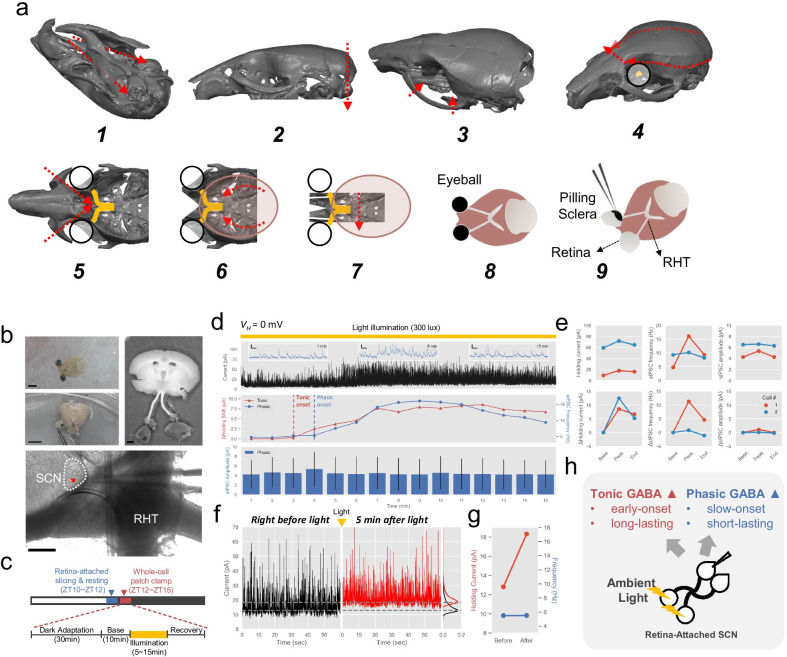
Direct evidence of light-triggered tonic GABA signaling in SCN via retina-attached slice patch. **a** Sequence of optimized retina-attached brain extraction method. Numbers indicate sequential steps of prep. **b** Eye or retina attached brain. Scale bars: 5 mm (top left). Retina attached SCN slice. Scale bar: 1 mm (top right). Retina-attached SCN slice fixed with anchor and accessed with patch pipette. Red asterisk denotes the position of patched cell. Scale bar: 200 $\mu$m; SCN is indicated with white dotted line (lower) **c** Timeline of experiment. Blue box represent retina attached slicing and resting time and red box represent whole-cell patch clamp periods (top). Magnified whole-cell recording protocol with light illumination highlighted with yellow box (lower). **d** Raw trace of voltage clamping (Holding potential, 0 mV) during ambient light illumination from cell#1. Insets, high time resolution trace at 1 min, 8 min, 15 min. Inset scale: 200 ms, 10 pA (top). 1 min bin analysis of phasic GABA (blue, sIPSC frequency) and tonic GABA (red, holding current shift) from cell #1 (middle). 1 min bin analysis of sIPSC amplitude from cell #1 (bottom), mean and error of 0.95 confidence intervals. **e** Paired point plots of light-triggered GABA signaling from two cells in SCN: holding current (top left), sIPSC frequency (top middle), sIPSC amplitude (top right), relative holding current change compared to baseline (bottom left), relative sIPSC frequency change compared to baseline (bottom middle), relative sIPSC amplitude change compared to baseline (bottom right). “Base” is first bin of light illumination; “Peak” is bin of maximum value; “End” is last bin of light illumination. **f** Representative raw 1 min traces of cell#1, right before the light illumination start (left, black), 5 min after light termination (middle, red) and data point distribution for measurement of shifted holding current (right). Dashed lines indicate mode of histograms. **g** Comparison of before and after holding GABA current (red) and phasic sIPSC frequency (blue) of cell#1. **h** Graphical description of light-induced GABA signaling in SCN

According to our tonic GABA profiling result, the level of extracellular GABA level gradually decreased along with the elapsed time after slicing. The fact that RHT is inevitably removed by dissection procedure, SCN cannot receive external light after slicing. Indeed, it has been previously reported that sustained GABA signaling has been linked with light-induced changes [3, 6, 26]. Therefore, we raised the hypothesis that the decrease in tonic GABA current is due to the loss of continuous light input through RHT. To test this hypothesis, we adopted the previously described retina-attached SCN slice preparation method [17] to see whether light can directly trigger tonic GABA signaling. By optimizing the retina-attached SCN extraction protocol (Fig. 2a) and combining NMDG-based protective method, we were able to obtain a healthy retina-attached SCN slices (Fig. 2b). To examine the effect of the light in SCN, we designed a protocol which mimics photo stimulation of phase delay (Fig. 2c). Although we report only two successful cases due to the technically challenging procedure, we showed, for the first time, the experimental evidence of light-triggered GABA signaling in SCN. After patching the ventrally located neurons in SCN, light illumination on retina triggered the early-onset of tonic GABA signaling ($\Delta$Holding current onset, t = 1.75 ± 1.25 min), followed by a slow-onset increase in the phasic GABA signaling (sIPSC frequency onset, t = 3.75 ± 0.25 min; n = 2; mean ± s.e.m) (Fig. 2d middle, Additional file 1: Fig. S4a, b). When sIPSC freqeucny was at the peak, we observed burst synaptic inputs (Fig. 2d top, insets). However, sIPSC amplitude of individual events did not vary during light illumination (Fig. 2d bottom, Additional file 1: Fig. S4b bottom), indicating that increase in tonic current is not due to experimental condition change, such as improvement of seal resistance. Moreover, tonic holding currents showed relatively sustained activation during light illumination ($\Delta$Holding current peak, 10.53 ± 1.98; end, 5.95 ± 0.77, end-to-peak percent, 56.5 %; n = 2) compared to phasic sIPSC frequency ($\Delta$sIPSC frequency peak, 6.07 ± 5.27; end, 1.75 ± 2.88, end-to-peak percent, 26.24 %; n = 2) (Fig. 2e). Especially, in one of two cells, we observed that tonic holding current remain elevated even after 5 min after termination of 15 min light illumination, whereas sIPSC frequency completely returned to original (before light illumination) frequency level (Fig. 2f, g). This result provides the first evidence that external light cue can directly trigger both tonic and phasic GABA signaling in SCN cell (Fig. 2h).

## Discussion

In this study, we have described the spatio-temporal profiling of tonic GABA signaling in SCN. As a result, the neurons with large tonic current were frequently found in dorsal SCN, with high correlation of sIPSC frequency. This finding is consistent with the previous finding [15]. Interestingly, slice-to-patch time interval was negatively correlated with tonic current size, suggesting a critical role of retinal light exposure in intact brain for generation of tonic GABA current in SCN. Therefore, we tested this hypothesis by retina-attached SCN slice patch clamping. As expected, light-illumination on retina directly triggered the GABA signaling in SCN. With this findings, we propose a novel mechanism of light-mediated circadian rhythm control in SCN involving tonic and phasic GABA signalings.

### Dorsal SCN bridges tonic GABA and excitatory GABA

We found that cells with high level of tonic GABA current are usually located at dorsal shell of SCN (Fig. 1h). To our knowledge, we are the first to show the distribution pattern of tonic GABA current in SCN (Fig. 1g). This dorsal shell-like distribution pattern of tonic GABA in SCN is intriguing because it can be directly linked with excitatory actions of GABA [5, 7, 24]. Previous modeling result predicted that the cells which are always excited by GABA release tonic GABA when they become depolarized [7]. If this is biologically feasible, the dorsal SCN neurons may exhibit reciprocal positive feedback loop between tonic and phasic GABA. Once cell depolarizes enough to release tonic GABA, the volume transmission to nearby neurons will significantly increase the excitability by excitatory tonic action of GABA [5, 7]. This positive feedback loop may result in long-lasting depolarization in dorsal SCN without any extra inputs once it is triggered. It would be interesting to investigate whether excitatory tonic GABA current exists in dorsal shell neurons and plays important role in synchronizing circadian rhythm.

### Relationship between tonic GABA and phasic GABA

Phasic GABA plays important role when precise sub-second level timing is important between neurons [27]. Although tonic GABA is not suitable for sub-second level control of neural activity, the amount of charge transfer is much bigger in tonic than phasic GABA [27]. As circadian rhythm modulation requires transcription level change, time scale involved in this process ranges at supra-minute level. Therefore, sustained tonic GABA signals may exert more powerful influence on SCN compared to transient phasic GABA signals [7]. Both phasic and tonic GABA signaling exhibit diurnal rhythm in SCN [15], but oscillation of tonic GABA is thought to be a consequence of the action-potential-mediated spill-over from synaptic GABA release. Our results of high correlation between sIPSC frequency and tonic current is consistent with previous finding, supporting the spill-over origin theory (Fig. 1c) [15]. In contrast, in our retina-attached SCN slice patch recordings, the onset of tonic GABA preceded that of phasic GABA, raising another possibility that extracellular GABA can originate from something other than spill-over [6, 10, 28]. Other sources include channel-mediated release of tonic GABA from nearby astrocytes, as it has been previously reported in the cerebellum, hippocampus, and thalamus [10–12, 14] However, this interesting possibility awaits future investigations.

### Relationship between phase delay and light triggered GABA signaling in SCN

Sustained activation of GABA$_A$ receptor signaling in SCN appears to be both necessary and sufficient to mediate light-induced phase delays of the circadian pacemaker [3, 6, 26]. However, how the brief light exposure (15 min) can efficiently shift the phase (several hours) of circadian oscillation via sustained GABA signaling has been a mystery. In the current study, we were able to show the the relationship between GABA and light stimulation (Fig. 2c). Considering the fact that tonic current remains elevated whereas sIPSC frequency returns to the original level after the light stimulation (Fig. 2d, e), sustained GABA signaling after light exposure in SCN is probably mediated by the form of tonic but not phasic. What can be inferred from this result is that the upstream source of long-lasting tonic and short-lasting phasic GABA might be different due to contrasting dynamics. One possibility is that the light-mediated tonic GABA originates not from the spill-over but from the direct release of astrocytic GABA [10]. Considering the fact that astrocyte can release tonic GABA non-synaptically by direct calcium elevation [14], the sustained tonic GABA signaling may originate from the light-induced astrocytic calcium signaling upon activation of G$_q$-coupled GPCR signaling. In other words, it means that light stimulation does not only enter the ventral region locally, but can also irritate the dorsal region.

### Involvement of glutamate signaling in light-triggered GABA response

Among various neurotransmitters, glutamate and PACAP are major molecules co-stored in RHT terminal [29]. Of these molecules, glutamate has been widely investigated as a light signal mediator in SCN [30]. If the light-induced GABA signaling is triggered by glutamate release from RHT terminal, investigation of how glutamate signaling causes GABA signaling in SCN neuron can broaden our understanding of the mysterious entrainment mechanism. Interestingly, retinal projections of intrinsically photosensitive retinal ganglion cells (ipRGCs) are densely innervating not only the ventral but also dorsal region of SCN [31], giving a possibility that light-mediated release from RHT terminals can universally affect SCN neurons. This finding opposes the conventional notion that dorsal shell SCN neurons are indirectly activated by SCN ventral core neurons under light stimulation. Thus, different light-mediated responses in SCN neurons seem to be caused by the receptor level heterogeneity (expression pattern of glutamate or PACAP receptors) but not the release level heterogeneity (region specific RHT innervation). Indeed, NR2C, a subunit of ionotropic NMDA receptor, has been shown to be selectively expressed in dorsal SCN region [32]. This study has shown that the astrocytic glutamate release triggers GABA release from SCN neurons during internal clock oscillation through NR2C. In contrast, our study suggests that the opposite mechanism is also possible when external light-cue is given; glutamate from the neurons (ipRGCs), may trigger GABA release from the astrocytes in SCN. Although there are indirect lines of evidence that astrocytes respond to glutamate in SCN [33, 34], it is not yet validated whether astrocytes in SCN indeed respond to the glutamate released upon external light. If this is the case, it would be interesting to further investigate which type of glutamate receptor(metabotropic or ionotropic) [30, 35] is involved in the light-triggered tonic GABA release.

### Source of ambient GABA in SCN

The cellular level source of tonic GABA can be either from the GABAergic neurons or the GABAergic astrocytes in the SCN. In terms of release mechanism of ambient GABA level, vesicle mediated spillover, astrocytic Best1 channel or reverse mode of GABA transporters are well known candidates [28]. It is well established that most of SCN neurons are GABAergic neurons [36, 37]. In contrast, it is not clear whether SCN astrocytes are GABAergic, which needs future validation. GATs are also involved in modulation of SCN tonic GABA currents [38], but shown to be expressed in both neurons and astrocytes [28]. Whether neuronal or astrocytic source or both, the release mechanism is most likely to be through the GABA-permeable Best1 channel [10]. These possibilities should be tested in future explorations.

In summary, we expect that the novel concepts and tools that we have developed in our study will shed the light on the veiled mechanisms of the light-induced entrainment.

## Supplementary information

**Additional file 1: Figure S1.** Assignment of relative horizontal and vertical coordinates fromSCN center.** Figure S2.** Tonic GABA grouped analysis result.** Figure S3.**Statistical comparison of cells grouped by size of tonic current.** Figure S4.**Light-triggered tonic GABA signaling in SCN cell #2.

**Additional file 2.** Raw data regarding spatiotemporal characterization of 76 cells in SCN.

## Data Availability

All data generated or analyzed during this study are included in this article.

## Abbreviations

SCN: Suprachiasmatic nucleus
GABA: $\gamma$-aminobutyric acid
sIPSC: spontaneous inhibitory postsynaptic current
