# Supplementary material for: Retina-attached slice recording reveals light-triggered tonic GABA signaling in suprachiasmatic nucleus
Source: Mol Brain. 2021 Nov 27;14:171. doi: 10.1186/s13041-021-00881-9 (PMC8626980; doi:10.1186/s13041-021-00881-9)
Supplement: Supplementary file 1 — Additional file 1: Figure S1. Assignment of relative horizontal and vertical coordinates fromSCN center. Figure S2. Tonic GABA grouped analysis result. Figure S3.Statistical comparison of cells grouped by size of tonic current. Figure S4.Light-triggered tonic GABA signaling in SCN cell #2. [file 13041_2021_881_MOESM1_ESM.pptx]

## Slide 1
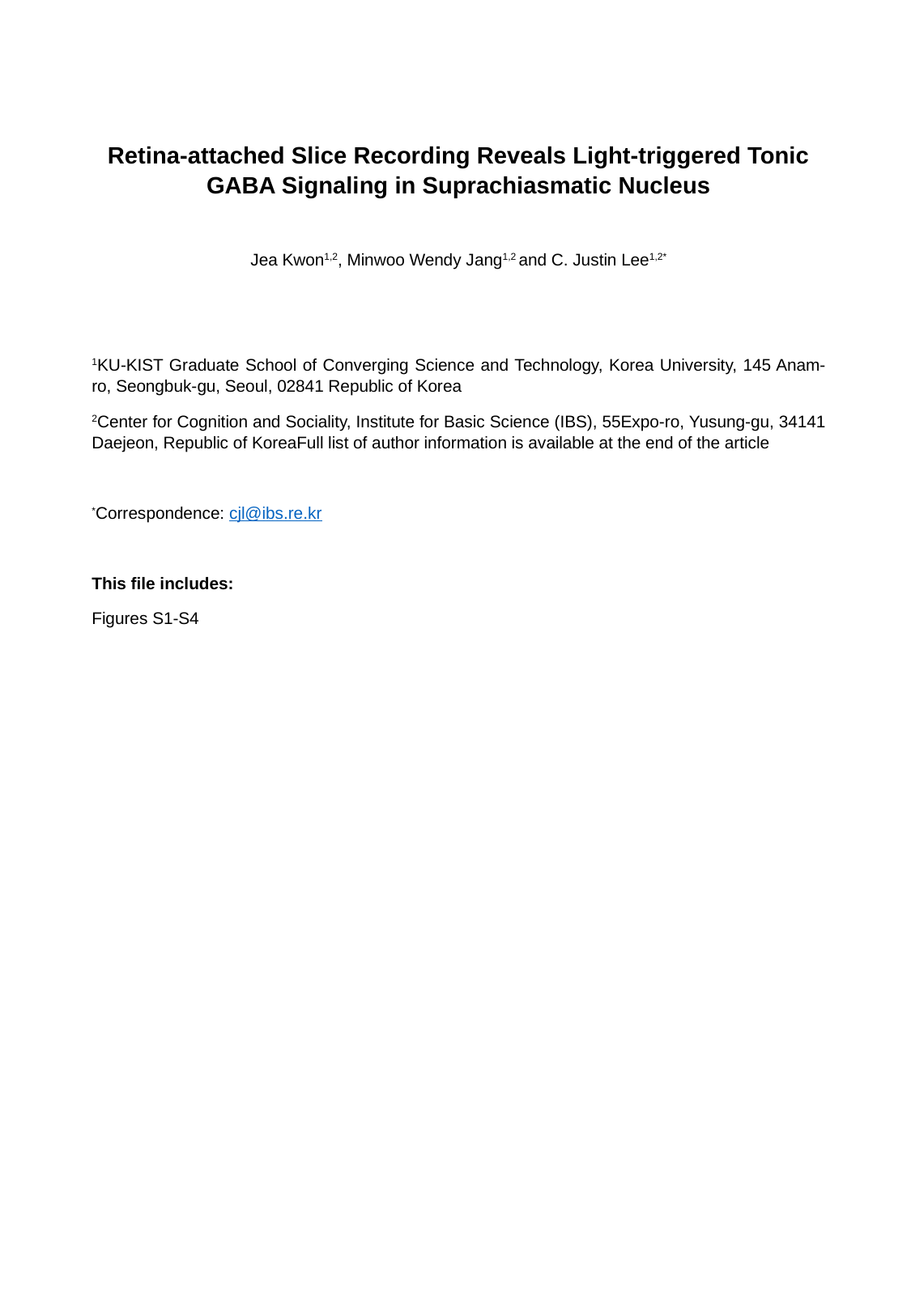

Retina-attached Slice Recording Reveals Light-triggered Tonic GABA Signaling in Suprachiasmatic Nucleus
Jea Kwon1,2, Minwoo Wendy Jang1,2 and C. Justin Lee1,2*
1KU-KIST Graduate School of Converging Science and Technology, Korea University, 145 Anam-ro, Seongbuk-gu, Seoul, 02841 Republic of Korea
2Center for Cognition and Sociality, Institute for Basic Science (IBS), 55Expo-ro, Yusung-gu, 34141 Daejeon, Republic of KoreaFull list of author information is available at the end of the article
*Correspondence: cjl@ibs.re.kr
This file includes:
Figures S1-S4

## Slide 2
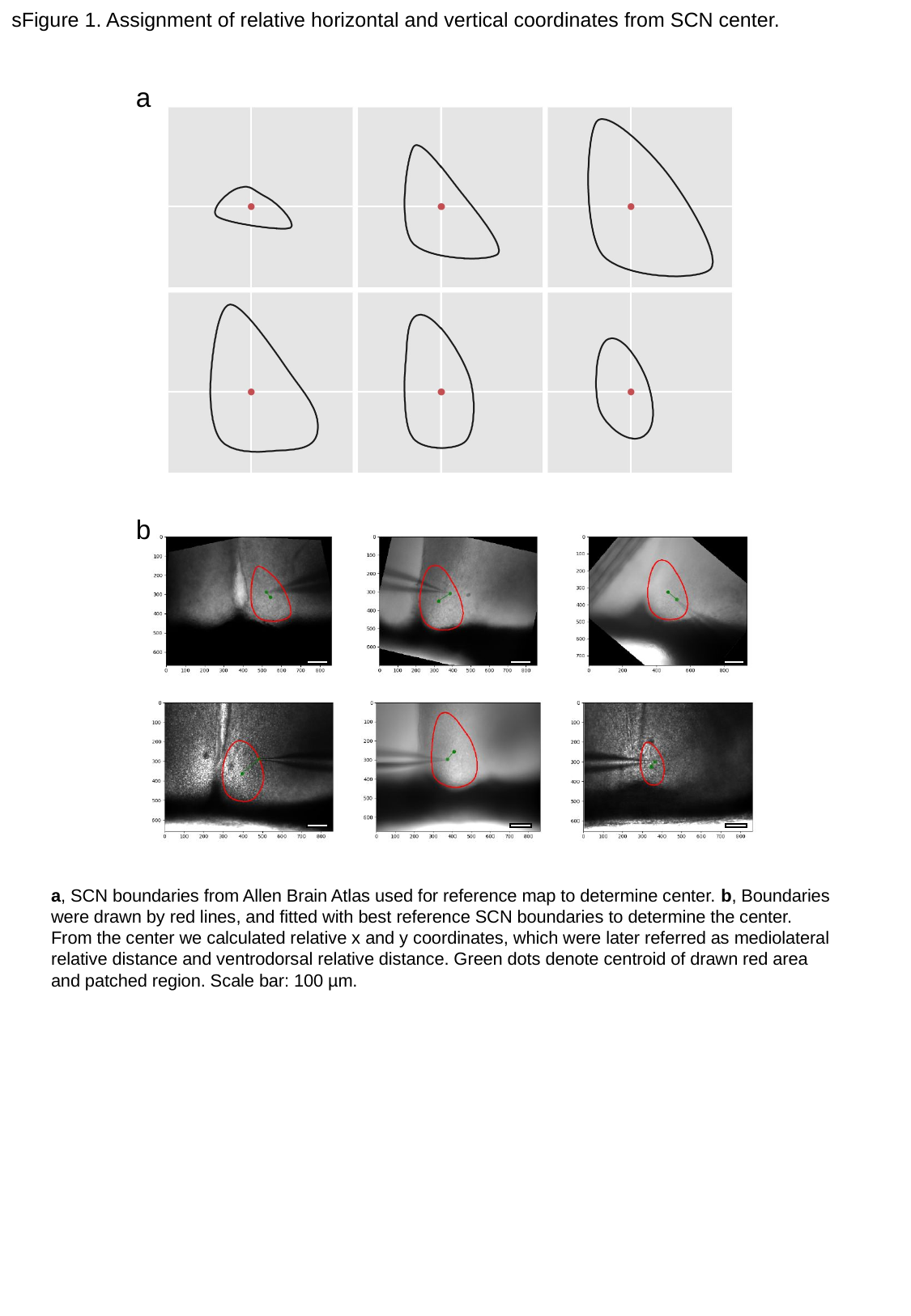

sFigure 1. Assignment of relative horizontal and vertical coordinates from SCN center.
a
b
a, SCN boundaries from Allen Brain Atlas used for reference map to determine center. b, Boundaries were drawn by red lines, and fitted with best reference SCN boundaries to determine the center. From the center we calculated relative x and y coordinates, which were later referred as mediolateral relative distance and ventrodorsal relative distance. Green dots denote centroid of drawn red area and patched region. Scale bar: 100 µm.

## Slide 3
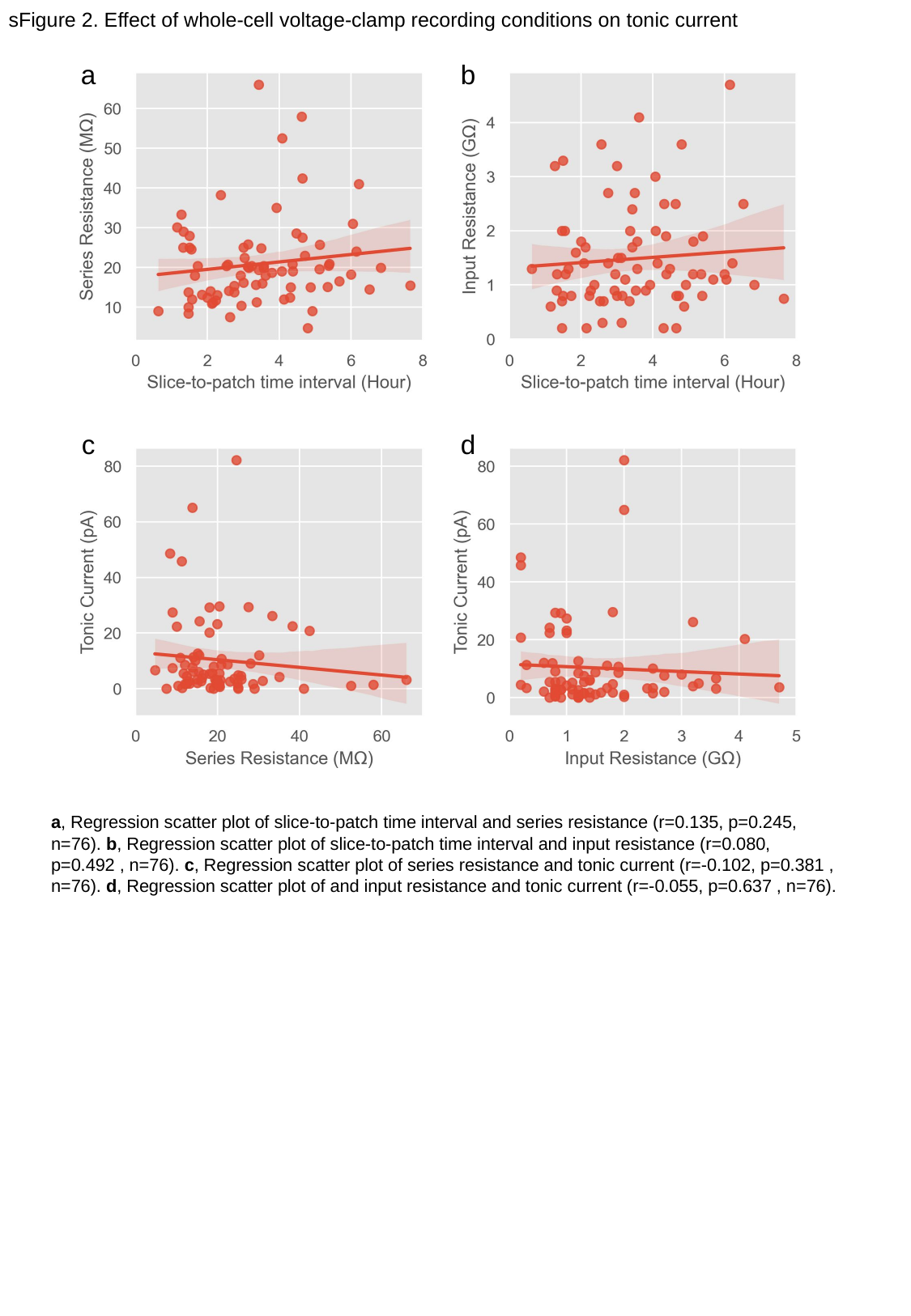

sFigure 2. Effect of whole-cell voltage-clamp recording conditions on tonic current
a
b
c
d
a, Regression scatter plot of slice-to-patch time interval and series resistance (r=0.135, p=0.245, n=76). b, Regression scatter plot of slice-to-patch time interval and input resistance (r=0.080, p=0.492 , n=76). c, Regression scatter plot of series resistance and tonic current (r=-0.102, p=0.381 , n=76). d, Regression scatter plot of and input resistance and tonic current (r=-0.055, p=0.637 , n=76).

## Slide 4
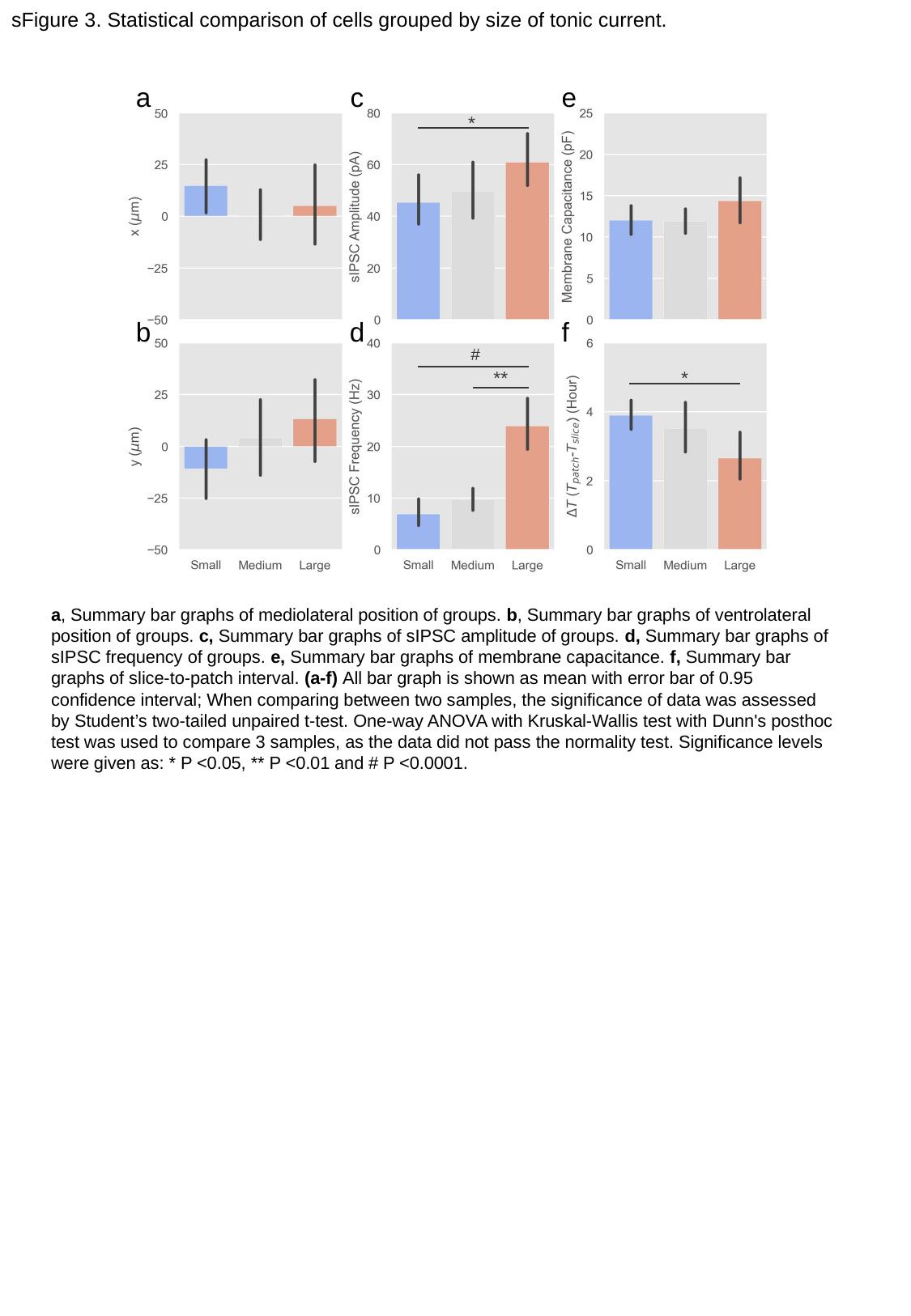

sFigure 3. Statistical comparison of cells grouped by size of tonic current.
a
c
e
*
#
**
*
b
d
f
a, Summary bar graphs of mediolateral position of groups. b, Summary bar graphs of ventrolateral position of groups. c, Summary bar graphs of sIPSC amplitude of groups. d, Summary bar graphs of sIPSC frequency of groups. e, Summary bar graphs of membrane capacitance. f, Summary bar graphs of slice-to-patch interval. (a-f) All bar graph is shown as mean with error bar of 0.95 confidence interval; When comparing between two samples, the significance of data was assessed by Student’s two-tailed unpaired t-test. One-way ANOVA with Kruskal-Wallis test with Dunn's posthoc test was used to compare 3 samples, as the data did not pass the normality test. Significance levels were given as: * P <0.05, ** P <0.01 and # P <0.0001.

## Slide 5
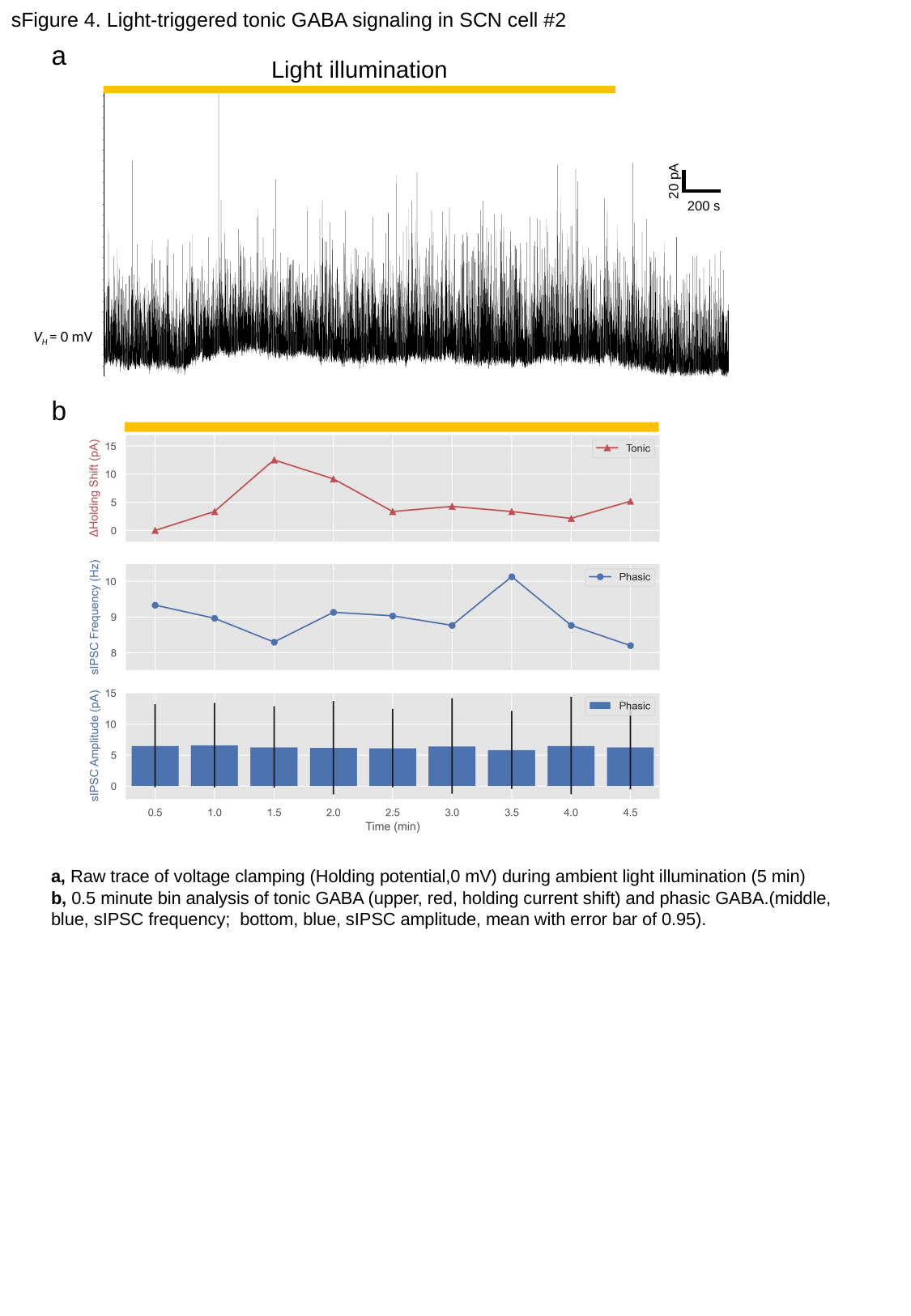

sFigure 4. Light-triggered tonic GABA signaling in SCN cell #2
a
Light illumination
20 pA
200 s
VH = 0 mV
b
a, Raw trace of voltage clamping (Holding potential,0 mV) during ambient light illumination (5 min)
b, 0.5 minute bin analysis of tonic GABA (upper, red, holding current shift) and phasic GABA.(middle, blue, sIPSC frequency; bottom, blue, sIPSC amplitude, mean with error bar of 0.95).
